# Impact of Policy and Funding Decisions on COVID-19 Surveillance Operations and Case Reports — South Sudan, April 2020–February 2021

**DOI:** 10.15585/mmwr.mm7022a3

**Published:** 2021-06-04

**Authors:** Talya Shragai, Aimee Summers, Olu Olushayo, John Rumunu, Valerie Mize, Richard Laku, Sudhir Bunga

**Affiliations:** ^1^Epidemic Intelligence Service, CDC; ^2^CDC COVID-19 Response Team; CDC; ^3^Division of Global Health Protection, Center for Global Health, CDC; ^4^Country Office, Juba, South Sudan, WHO; ^5^Ministry of Health, Juba, South Sudan; ^6^Country Office, Juba, South Sudan, Division of Global HIV & TB, Center for Global Health, CDC.

Early models predicted substantial COVID-19-associated morbidity and mortality across Africa (*1*–*3*). However, as of March 2021, countries in Africa are among those with the lowest reported incidence of COVID-19 worldwide (*4*). Whether this reflects effective mitigation, outbreak response, or demographic characteristics, (*5*) or indicates limitations in disease surveillance capacity is unclear (*6*). As countries implemented changes in funding, national policies, and testing strategies in response to the COVID-19 pandemic, surveillance capacity might have been adversely affected. This study assessed whether changes in surveillance operations affected reporting in South Sudan; testing and case numbers reported during April 6, 2020–February 21, 2021, were analyzed relative to the timing of funding, policy, and strategy changes.* South Sudan, with a population of approximately 11 million, began COVID-19 surveillance in February 2020 and reported 6,931 cases through February 21, 2021. Surveillance data analyzed were from point of entry screening, testing of symptomatic persons who contacted an alert hotline, contact tracing, sentinel surveillance, and outbound travel screening. After travel restrictions were relaxed in early May 2020, international land and air travel resumed and mandatory requirements for negative pretravel test results were initiated. The percentage of all testing accounted for by travel screening increased >300%, from 21.1% to 91.0% during the analysis period, despite yielding the lowest percentage of positive tests among all sources. Although testing of symptomatic persons and contact tracing yielded the highest percentage of COVID-19 cases, the percentage of all testing from these sources decreased 88%, from 52.6% to 6.3% after support for these activities was reduced. Collectively, testing increased over the project period, but shifted toward sources least likely to yield positive results, possibly resulting in underreporting of cases. Policy, funding, and strategy decisions related to the COVID-19 pandemic response, such as those implemented in South Sudan, are important issues to consider when interpreting the epidemiology of COVID-19 outbreaks.

COVID-19 surveillance in South Sudan is operated by the South Sudan Ministry of Health (MOH) with support from implementing partners. The surveillance system collected testing and case data from five sources:^†^ 1) screening of inbound travelers at points of entry, 2) rapid response team testing of persons with COVID-19–compatible symptoms who called an alert hotline (alert), 3) contact tracing, 4) testing of symptomatic persons seeking health care for any reason (sentinel surveillance), and 5) screening of persons before outbound travel (travel screening). Symptomatic persons were tested by alert and sentinel surveillance testing; persons with a known exposure were tested through contact tracing, point of entry surveillance and travel screening tested asymptomatic persons with no known exposure. Testing was conducted free of charge at the National Public Health Laboratory (NPHL), at public mobile laboratories, or at private laboratories (in which testing costs were approximately $40–$150 USD per test).Testing was performed on oropharyngeal or nasopharyngeal swab specimens using reverse transcription–polymerase chain reaction. Surveillance staff members completed a paper form upon specimen collection, which was attached to the laboratory results and physically transported or emailed from the testing laboratory to the Public Health Emergency Operations Center in Juba, South Sudan, by the MOH and supported by implementing partners,^§^ for entry into the central COVID-19 surveillance database.

During the course of the COVID-19 pandemic in South Sudan, national-level changes affected travel (including travel restrictions and travel testing requirements), testing strategies, funding and logistical support, and laboratory capacity. For this analysis, information on these changes was obtained through interviews with national-level personnel and review of published documents.^¶ ^Temporal trends in the weekly number tests for SARS-CoV-2 , the virus that causes COVID-19, performed and the percentage of tests with positive results were analyzed, based on the result reporting date. Results were examined at the national level and by surveillance source before, during, and after major policy, strategy, and funding changes that affected surveillance procedures and practices. Records with missing specimen collection date or surveillance source were excluded. The surveillance source variable was standardized across records.** This activity was reviewed by CDC and conducted consistent with applicable federal law and policy.^††^

Among 101,021 COVID-19 tests performed during April 6, 2020–February 21, 2021, a total of 99,533 (98.5%) were included in this analysis; the remainder were excluded because of missing data. Overall, 6,766 (6.8%) tests yielded positive results for SARS-CoV-2. The number of weekly tests peaked three times: during the week beginning May 25, 2020 (2,423 tests [2.4%] ), the week beginning November 2, 2020 (4,767 tests [4.8%]), and the week beginning February 15, 2021 (6,031 tests [6.1%]), which is the last week for which data were available (Figure 1). The percentage of tests yielding positive results first peaked during the first week of June 2020 (537 of 1,668 [32.2%] positive), and again the week beginning February 15, 2021 (1,385 of 6,031 [22.5%] positive). Among all 99,533 tests, 78,146 (78.5%) were from travel screening (4,559 [5.8%] positive), 3,742 (3.8%) were collected as part of contact tracing (961 [25.7%] positive), 3,224 (3.2%) were from alerts (695 [21.6%] positive), 11,443 (11.5%) were from point of entry screening (256 [2.2%] positive), and 2,978 (3.0%) were from sentinel sites (295 [9.9%] positive).

**FIGURE 1:**
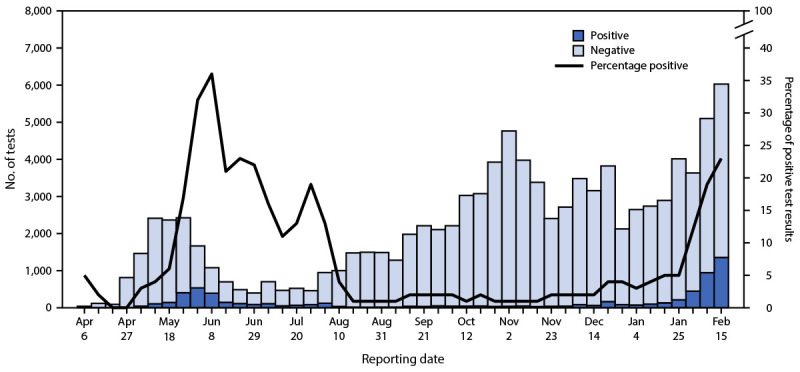
COVID-19 test results, by test reporting* date (N = 99,553) — South Sudan, April 6, 2020–February 21, 2021 * Surveillance data analyzed were from point of entry screening, testing of symptomatic persons who contacted an alert hotline, contact tracing, sentinel surveillance, and outbound travel screening.

A number of policy, strategy, and funding changes affected COVID-19 surveillance in South Sudan during the project period (Table). Travel screening testing increased after domestic and international travel restrictions were relaxed and travel testing requirements began in mid-May 2020 (Figure 2). Travel screening initially decreased after testing requirements for domestic travel were relaxed in late May 2020, but increased after travel restrictions were further relaxed through August 2020. Travel screening again decreased in early December 2020 after testing transitioned from the NPHL to private laboratories, followed by an increase later in the month after data sharing agreements were established between private laboratories and the MOH. During the week beginning February 15, 2021, travel screening testing represented 90.1% of all testing, an increase of >300% from June 2020, when it represented 21.1% of testing (Supplementary Figure, https://stacks.cdc.gov/view/cdc/106331). 

**TABLE:** Policy, strategy, and funding changes affecting COVID-19 surveillance operations, by surveillance source and date of change — South Sudan, April 2020–January 2021

Source/Date*	Policy, strategy, or funding change
**Travel screening surveillance**	
Mar 24, 2020	International borders were closed to passenger travel; domestic travel ban imposed soon after.
May 11, 2020	International and domestic travel bans were lifted.
May–Aug 26, 2020	Requirement of negative test certificate before domestic travel was relaxed in May and ended in August.
Jul 9, 2020	Regularly scheduled passenger travel resumed at Juba International Airport.
Oct 1–15, 2020	Ugandan land border was opened for passenger travel.
Dec 5, 2020	Travel screening was transferred to a private laboratory.
Dec 28, 2020	Data sharing agreements between private laboratories and South Sudan MOH were enacted.
Jan 18, 2021	A second private laboratory was opened (cost = $40–$150 per test).
**Contact tracing surveillance**	
Jul 2020	Contact testing strategy was changed from testing all contacts to testing only symptomatic contacts or contacts at increased risk of adverse outcomes.
Sep 2020	Contact tracing program activities were transferred to a new organization.
Jan 4, 2021	Policy to test all contacts, symptomatic and asymptomatic, was reinstated.
**Alert surveillance**	
Jul–Sep 2020	Funds and logistical support were reduced for the rapid response teams and alert hotline system.
**Points of entry surveillance**	
Jun 2020	National laboratory testing of most samples shipped from points of entry was discontinued because of limited testing capacity.
Jul 25, 2020	Mobile laboratory established at Nimule border crossing with Uganda began data sharing with South Sudan MOH.
**Sentinel site surveillance**	
May 2020	Forty-five health facilities were enlisted for the sentinel site surveillance system.
Jan 1, 2021	Number of sentinel sites were reduced to 18.
Jan 14, 2021	Number of sentinel sites were reduced to three.

**Abbreviation**: MOH = Ministry of Health.

* Dates are specified to the day if the exact date or range of dates is known, or to the month and year when exact date or range of dates is unknown.

**FIGURE 2:**
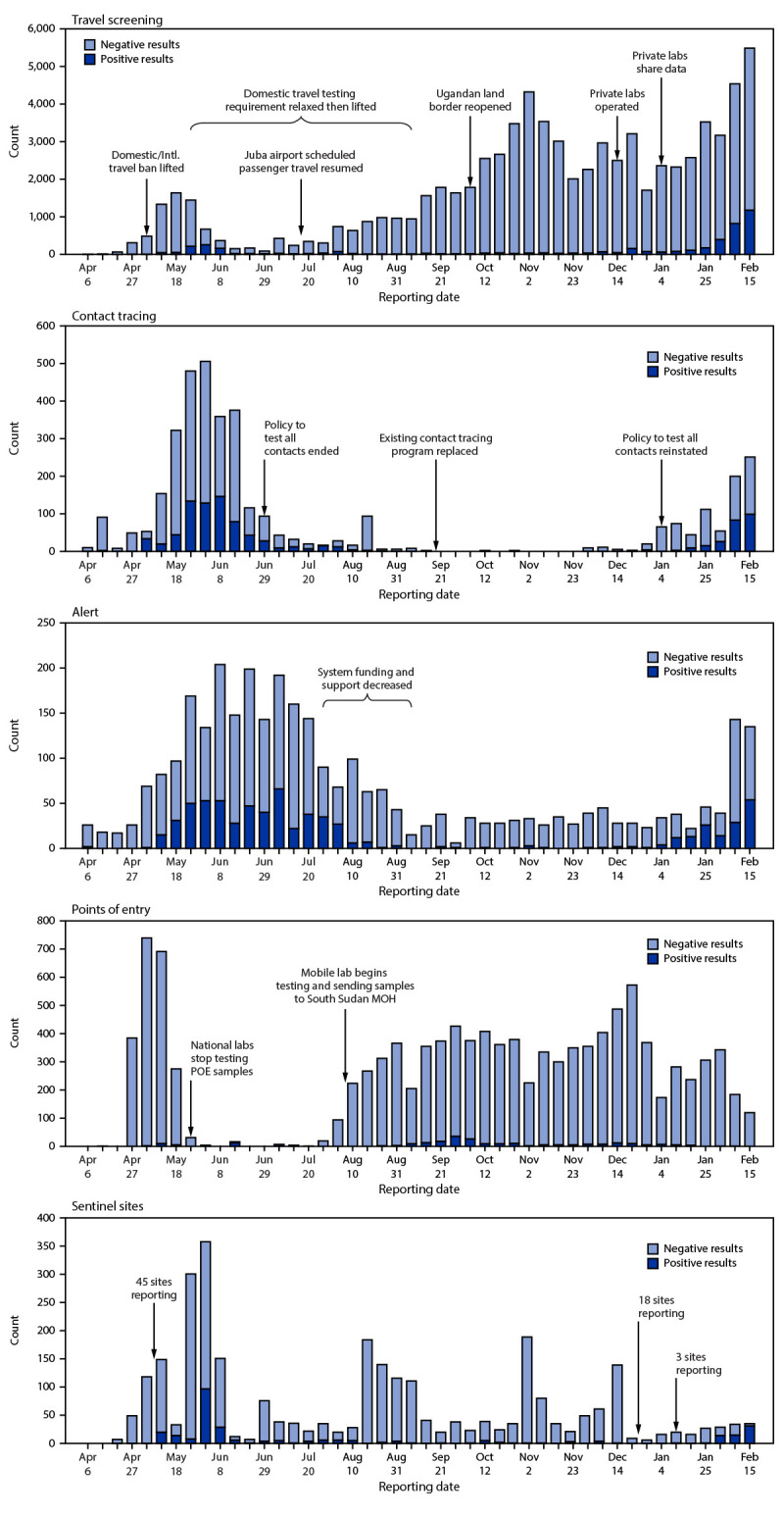
Number* and results of COVID-19 tests, by surveillance source,^†^ and major policy and funding changes correlated with changes in testing/positive case counts — South Sudan, April 6, 2020**–**February 21, 2021 **Abbreviations:** MOH = Ministry of Health; POE = point of entry. * Y-axes scaled differently in each panel. † Travel screening tested outbound travelers. Contact tracing tested those with a known exposure to a confirmed positive case. Alert testing consisted of rapid response teams testing persons with COVID-19–compatible symptoms who called the COVID-19 alert hotline. Point of entry screening tested persons as part of screening during inbound travel. Sentinel site surveillance was conducted at health facilities and tested persons who sought care for any reason and were experiencing COVID-19–compatible symptoms.

In July 2020, reductions in funding and logistical support for the alert and contact tracing systems occurred, and the national contact testing strategy changed from recommending testing of all contacts to testing only symptomatic persons or those considered to be at increased risk for adverse outcomes. After this change, the percentage of testing through contact tracing and alerts declined from 52.6% in June 2020 to 3.4% in January 2021 (Table) (Figure 2) (Supplementary Figure, https://stacks.cdc.gov/view/cdc/106331). In early January 2021, the policy to test all contacts irrespective of symptoms was reinstated, and although subsequent contact tracing and alert testing increased, these sources represented just 6.3% of testing during the week beginning February 15, 2021.

During the week beginning May 5, 2020, point of entry surveillance represented one half (50.6%) of all SARS-CoV-2 testing; however, because of limited resources, the NPHL discontinued testing these specimens, after which these specimens declined to <1% of all testing during June–July 2020 (Table) (Figure 2) (Supplementary Figure https://stacks.cdc.gov/view/cdc/106331). A mobile laboratory was established in late July at the Ugandan border and testing of specimens from points of entry subsequently increased and represented 2%–8% of all testing through the week beginning February 15, 2021.

Sentinel surveillance began with three sites in April 2020 and increased to 45 in May 2020. Tests from sentinel sites fluctuated during August 2020–January 2021, likely because of variations in weekly reporting rates, and decreased after the number of sites was reduced to 18 on January 1, 2021 and then to three later in the month (Figure 2) (Supplementary Figure, https://stacks.cdc.gov/view/cdc/106331).

## Discussion

COVID-19 data can be better understood in the context of a country’s funding, policy, and strategy changes. In South Sudan, testing through alert and contact tracing decreased after changes in policy, strategy, and funding affected those programs, which are typically associated with high percentages of positive test results. Changes in travel policies drove increased demand for travel screening, which, in February 2021, accounted for more than 90% of daily tests. Overall, testing increased in South Sudan over the project period, but shifted toward sources less likely to yield a positive result; outbound travel screening, which tested asymptomatic populations with no known exposure to a case, had the lowest overall yield of positive results throughout the project period. These changes might have resulted in substantial underreporting of positive cases.

Other African countries experienced a second wave of COVID-19 cases in early 2021, and in some, this has been linked to the more highly transmissible B.1.351 COVID-19 variant (*7*). Cases also increased in South Sudan during January–February 2021, from 73 cases during the first week of January to 1,358 during the week beginning February 15; however, because of inability to conduct genomic sequencing in-country and because official reported numbers likely underestimated cases, the scope of and reason for the surge in cases are not well understood. Accurate determination of SARS-CoV-2 transmission and COVID-19 disease incidence in South Sudan requires data-driven policies, funding, and logistic and human resource support for surveillance activities. Although travel-related testing that is low-yield and poorly targeted should take lower priority, the mandatory requirement of negative test results at a destination country imposes the need to prioritize travel testing in departure countries even in a resource constrained setting such as South Sudan. Policy decisions based on public health recommendations must ensure that testing focuses on higher-risk and higher-yield populations, not only to identify cases and better quantify the outbreak but to optimize the use of limited testing resources. 

The findings in this report are subject to at least six limitations. First, the relationship between policy, funding, and strategy and changes in testing and cases might not imply causality. In addition, availability of supplies might have limited testing capabilities at different timepoints. Second, this study does not account for competing priorities. Responses to other disease outbreaks, malnutrition, and major flooding in July 2020 might have diverted resources from COVID-19 surveillance.^§§^ Third, data collection methods, including categorization of surveillance source, varied over time; this analysis relied on several assumptions to standardize variables for comparison. Fourth, this analysis assumed that the surveillance source was correctly classified and that all testing was recorded, which could not be verified. Fifth, interpretation of surveillance and testing data is further limited by the absence of health care facility–level disaggregated data for comparison. Finally, SARS-CoV-2 diagnostic test numbers in this report are lower than those published by the South Sudan MOH (*8*) because records with missing specimen collection date or surveillance source were excluded from this analysis; however, the extent of exclusion was minimal (1.5%).

Interpretation of COVID-19 case reports and transmission patterns must be placed in geographic, temporal, resource, and policy context. For South Sudan, and possibly other countries where response funding, strategies, and policies have changed over time, surveillance data are likely driven by operational and resource realities rather than by transmission dynamics alone. Similarly, detailed analyses of outbreak data from other countries might help in understanding how policy decisions affect surveillance data, leading to more informed decisions about public health action.

**Summary**

**What is already known about this topic?**

As of March 2021, African countries have reported fewer COVID-19 cases than have countries in other regions. The extent to which this is due to surveillance limitations is unknown.

**What is added by this report?**

Policy, funding, and strategy changes in South Sudan influenced the number of SARS CoV-2 tests performed and the populations tested. Underreporting of testing rates and detected cases, including a February 2021 COVID-19 surge, might have occurred after policy changes led to an increase in travel screening of asymptomatic persons with no known contact with a positive case and a decrease in testing of suspected cases

**What are the implications for public health practice?**

Policy, funding, and strategy decisions related to the COVID-19 pandemic response, such as those in South Sudan, are important considerations when interpreting the epidemiology of COVID-19 outbreaks.

## Footnotes

* As of May 2021, South Sudan remains at Level 4 travel advisory (https://travel.state.gov/content/travel/en/traveladvisories/traveladvisories/south-sudan-travel-advisory.html). Most testing for SARS CoV-2 is poorly targeted and occurs largely among asymptomatic individuals as part of pretravel screening. Given the low testing rates and poorly targeted testing, the reported low incidence rate and case counts (which are dependent on testing volume) are not reflective of the actual magnitude of the outbreak.

† Points of entry included major airports and land borders (primarily the land border with Uganda). Alert surveillance also included postmortem testing of persons suspected to have died from COVID-19. Sentinel surveillance sites tested persons seeking health care for any reason who had symptoms of COVID-19. Outbound travel screening also included some asymptomatic persons tested for nontravel-related reasons. Surveillance source testing was available for all persons in South Sudan regardless of citizenship. All persons who tested positive were supported through case management programs that either support home-based care for asymptomatic, mild, or moderate cases or provided care at a dedicated COVID-19 medical facility for severe or critical cases.

§ South Sudan’s COVID-19 response is funded by donors, including the Bureau for Humanitarian Assistance, U.S. Agency for International Development; European Civil Protection and Humanitarian Aid Operations, European Commission; Foreign, Commonwealth & Development Office, Government of the United Kingdom; and CDC.

¶ Interviews were conducted in person, over the phone, or via email with national-level personnel coordinating the COVID-19 response and leading operations of each surveillance source. These persons were asked to identify any major funding, policy, or strategy changes that affected COVID-19 testing, to describe the impact on surveillance operations, and to provide published documentation of any identified changes as available. Documents reviewed were high level COVID-19 task force communications, weekly situation reports on COVID-19, South Sudan COVID-19 response guidelines and standard operating procedures, and daily updates from the South Sudan MOH, which were distributed via an email listserv and intermittently uploaded to the MOH website.

** In consultation with the South Sudan COVID-19 response data management unit, records that included “screening before travel,” “screening bef travel,” “screening,” and “screening to know status” were all categorized as travel screening; records with “alert,” “suspect,” and “suspected COVID-19” were all categorized as alert; and those with “POE” and “screening POE” were categorized as point of entry screening. All records were transformed to lower case, and all punctuation was removed to standardize differences in spelling. Any surveillance source with a Levenshtein distance (i.e., the minimum number of single-character edits between two words required to change one word into the other) of no more than two, excluding differences in capitalization and punctuation, was recategorized to the surveillance source that most closely matched the spelling after the standardization process.

†† 45 C.F.R. part 46.102(l)(2), 21 C.F.R. part 56; 42 U.S.C. Sect. 241(d); 5 U.S.C. Sect. 552a; 44 U.S.C. Sect. 3501 et seq.

§§ Information on other health events was received from the Public Health Emergency Operations Center of South Sudan (all-hazards update), presented at their weekly meeting, which compiles information on ongoing outbreaks and events affecting public health in South Sudan.

## References

[1] Massinga Loembé M, Tshangela A, Salyer SJ, Varma JK, Ogwell Ouma AE, Nkengasong JN. COVID-19 in Africa: the spread and response. Nat Med 2020;26:999–1003. 10.1038/s41591-020-0961-x 32528154

[2] Nkengasong JN, Mankoula W. Looming threat of COVID-19 infection in Africa: act collectively, and fast. Lancet 2020;395:841–2. Epub Feb 27, 2020. 10.1016/S0140-6736(20)30464-5 32113508 PMC7124371

[3] El-Sadr WM, Justman J. Africa in the path of Covid-19. N Engl J Med 2020;383:e11. Epub April 17, 2020. 10.1056/NEJMp2008193 32302075

[4] World Health Organization. Weekly epidemiological update–2 March 2021. Geneva, Switzerland: United Nations, World Health Organization; 2021. Accessed Mar 24, 2021. https://www.who.int/publications/m/item/weekly-epidemiological-update---2-march-2021

[5] Chitungo I, Dzobo M, Hlongwa M, Dzinamarira T. COVID-19: unpacking the low number of cases in Africa. Public Health in Practice 2020;1:100038. Epub Dec 22, 2020. 10.1016/j.puhip.2020.100038 34173573 PMC7485446

[6] Rutayisire E, Nkundimana G, Mitonga HK, Boye A, Nikwigize S. What works and what does not work in response to COVID-19 prevention and control in Africa. Int J Infect Dis 2020;97:267–9. 10.1016/j.ijid.2020.06.024 32535304 PMC7289731

[7] Regional Office for Africa, World Health Organization. New COVID-19 variants fueling Africa’s second wave. Geneva, Switzerland: United Nations, World Health Organization; 2020. https://www.afro.who.int/news/new-covid-19-variants-fuelling-africas-second-wave

[8] Republic of South Sudan Ministry of Health. Update on COVID-19 Response. Juba, South Sudan: Republic of South Sudan, Ministry of Health; 2021. Accessed March 18, 2021: https://moh.gov.ss/daily_updates.php

